# Novel RNA viruses in oysters revealed by virome

**DOI:** 10.1002/imt2.65

**Published:** 2022-11-29

**Authors:** Peng Zhu, Guang‐Feng Liu, Chang Liu, Li‐Ling Yang, Min Liu, Ke‐Ming Xie, Shao‐Kun Shi, Mang Shi, Jing‐Zhe Jiang

**Affiliations:** ^1^ Key Laboratory of South China Sea Fishery Resources Exploitation and Utilization, Ministry of Agriculture and Rural Affairs, South China Sea Fisheries Research Institute Chinese Academy of Fishery Sciences Guangzhou Guangdong China; ^2^ College of Marine Ecology and Environment Shanghai Ocean University Shanghai China; ^3^ One Health Biotechnology (Suzhou) Co., Ltd. Jiangsu China; ^4^ College of Life Science and Biopharmacy Guangdong Pharmaceutical University Guangzhou Guangdong China; ^5^ Ministry of Fisheries Technology Shenzhen Fisheries Development Research Center Shenzhen Guangdong China; ^6^ School of Medicine Sun Yat‐sen University Shenzhen Guangdong China

## Abstract

Eighteen novel RNA viruses were found in *Crassostrea hongkongensis*. Phylogenic analysis shows evidence of recombination between major genes of viruses. Picobirnaviruses are ubiquitous and abundant in oysters.

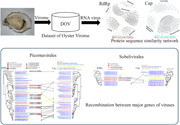

## INTRODUCTION

Oysters (phylum *Mollusca*, class *Bivalvia*, order *Pterioida*) are globally distributed shellfish and are an important marine biological resource that is available to humans. Oysters have high nutritional value and are the most farmed shellfish in the world. As the largest oyster producer, China produced 82,593,752 tons of oysters in 2019, accounting for 85.3% of the world's total output. Being filter feeders, oysters can filter up to 5 L of seawater through their gills every hour and enrich suspended microorganisms and particles by factors of a thousand to a hundred thousand times their seawater concentrations, making it easy for viruses to accumulate in oysters. Oysters have a clustered and sessile lifestyle and bring stable and lasting improvements to nearshore marine environments by, for example, reducing water turbidity and purifying water [1, 2]. However, oysters have evidently no acquired immune system [3], which may further increase the probability of virus transmission among oysters.

In 1972, Farley et al. found herpes virus infection in invertebrates in the United States, and showed that oyster deaths caused by the herpes virus were more common in high‐temperature conditions; the virus was named Ostreid herpesvirus‐1 (OsHV‐1) [4]. The mortality rate of OsHV‐1‐infected shellfish seedlings and young shellfish is >90%, which is very harmful to the oyster industry. In addition to OsHV‐1, other oyster‐associated viruses have been reported, including a *Papovaviridae* virus that causes oyster “Oocystitis,” which leads to egg and gamete cell hypertrophy, and gill necrosis virus, an *Iridoviridae* virus that may have been the main cause of mass death of the bivalve *Crassostrea angulata* population in the late 1960s [5, 6]. Moreover, *Togaviridae, Reoviridae*, and *Picornaviridae* viruses have also been reported in shellfish hosts [6]. Most of the studies on these viruses were confined to pathological and electron microscopic observations, and no in‐depth reports have been published so far. Norovirus, hepatitis A virus, and astrovirus have been found in farmed oysters, but these viruses are not pathogens of oysters [7]. Research progress on viruses that are pathogenic to oysters is still very slow; therefore, the identification of oyster pathogens is a top priority for oyster disease prevention and control.

With the development of high‐throughput sequencing technologies, methods such as viromics and meta‐transcriptomics have overcome the dependence of traditional virology studies on host cell culture and greatly improved the efficiency of the discovery and identification of new viruses in invertebrates [8]. For example, 1445 RNA viruses with complete genomes were found by transcriptome analysis of more than 220 invertebrate species from nine animal phyla [9], which greatly expanded the understanding of the virus community. Seven complete RNA virus genomes were obtained from *Crassostrea gigas* and *Mytilus galloprovincialis* host transcriptome data and classified as *Picornavirales*; six of them were new viruses [10]. Intracellular RNA libraries of California sea hare (*Aplysia californica*) and frog (*Microhyla fissipes*) were sequenced and the complete genomes of two novel viruses of *Nidovirales* were found [11]. A comparative study on healthy and infected starfish identified a suspected pathogen of *Parvoviridae* and confirmed that it was also widely present in plankton and marine sediments [12]. Genome fragments of 117 RNA viruses that contained RdRp genes distributed in nine viral families or orders were identified in 58 invertebrate species across three seas [13].

The Data set of Oyster Virome (DOV) was reported by Jiang et al. [14]. DOV, which contains 728,784 contigs (≥800 bp) of nonredundant virus operation taxa (vOTU) and 3473 high‐quality viral genomes, provided the first comprehensive description of oyster viral community structure. Among them, 4958 RNA virus‐related vOTUs were found to be particularly noteworthy [14]. This study used bioinformatics tools to analyze the genomes of 18 oyster‐associated RNA viruses among the RNA virus‐related vOTUs in DOV. The results provide an important reference for the expansion of the DOV and the identification of oyster viral pathogens.

## RESULTS

### Eighteen novel RNA viruses were found in oysters

**Figure 1:**
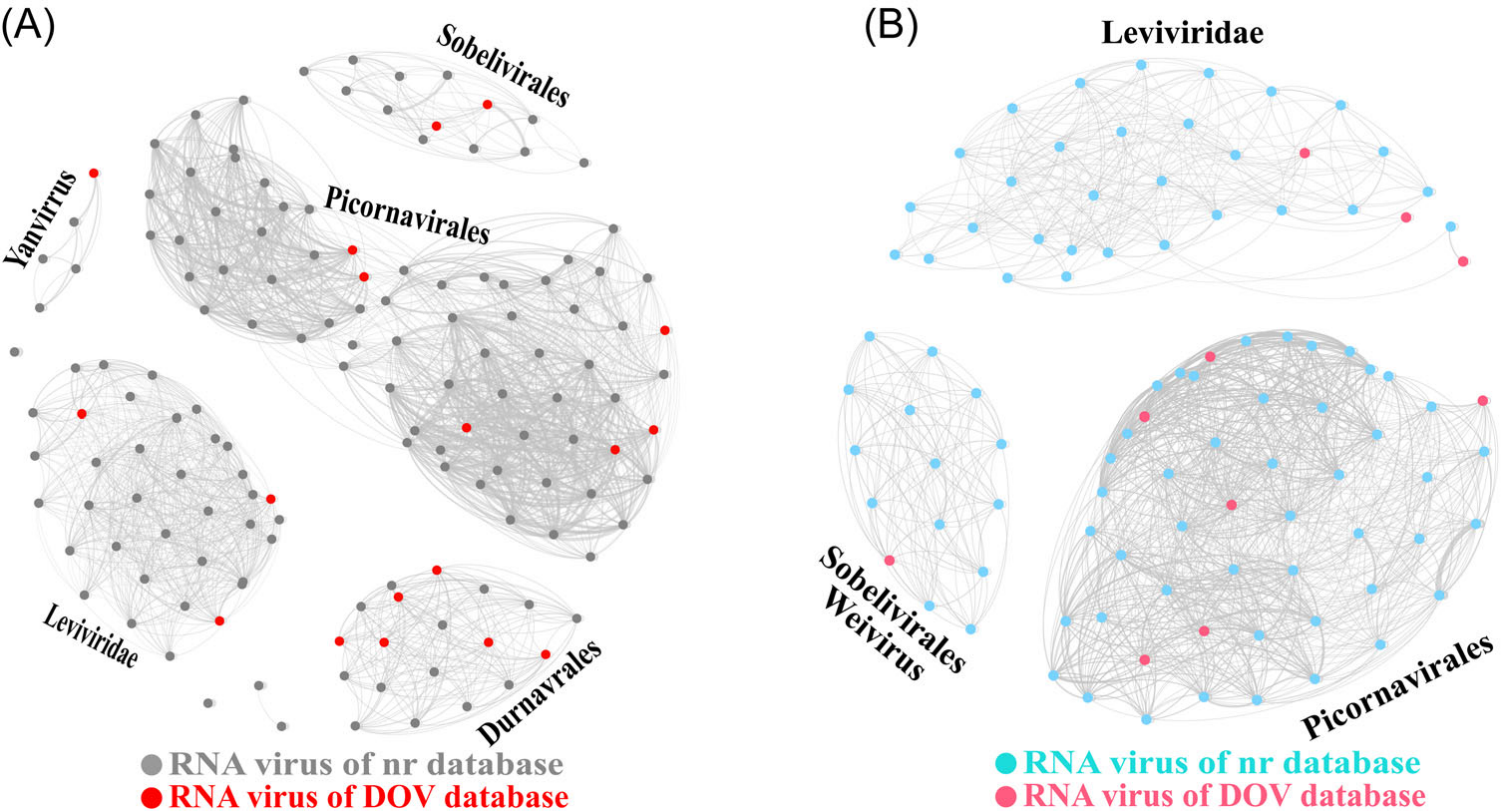
The variety of novel RNA viruses found in oysters. (A) Clustering network of 147 RdRp protein sequences. (B) Clustering network of 99 capsid protein sequences. The networks were visualized using the Fruchterman‐Reingold algorithm in Gephi (version 0.9.2). Dots represent different sequences. Edges indicate that the DIAMOND BLASTP scores between the connected dots were ≥57 (A) and ≥43.5 (B).

We used 18 RNA virus sequences from the DOV obtained previously [14] for deep analysis. Because the 18 RNA virus sequences were very different they could not be reliably aligned, and therefore could not be used to construct a unified and reliable phylogenetic tree. Therefore, we constructed a clustering network based on the similarity of the encoded RdRp and capsid protein sequences. The RdRp protein sequences of the 18 oyster‐associated RNA viruses and related viruses in the nr database clustered roughly into five groups (Figure 1A), which means they belonged to five families or orders (*Sobelivirales, Picornavirales, Leviviridae, Durnavirales*, and Yanvirus) (Supporting Information: Table S1). Only 10 of the 18 genomes were annotated with capsid proteins, which were clustered into three groups (Figure 1B, Supporting Information: Table S1) (*Sobelivirales*‐Weivirus, *Picornavirales*, and *Leviviridae*).

### Evidence of gene exchange among RNA viruses

**Figure 2:**
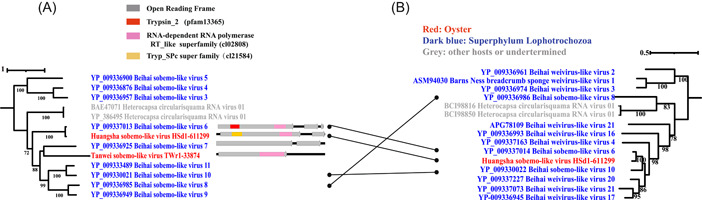
Phylogenic analysis shows evidence of recombination between major viral proteins. Phylogenetic trees of RdRp (A) and capsid proteins (B) of oyster‐associated *Sobelivirales*. The maximum likelihood phylogenetic tree was constructed using IQtree (version 2.1.4) with the sequences. ModelFinder was set as MFP and 1000 ultrafast bootstrap replicates were used. Bootstrap values >70 are shown. The domains in the genome structure were annotated using the NCBI Conserved Domain Database.

*Sobelivirales* are RNA viruses that are found in plants or invertebrates and have a sense, non‐segmented genome of 4‐4.6 kb [13, 15]. We found two oyster‐associated *Sobelivirales* viruses (Figures 1 and 2A). Huangsha sobemo‐like virus HSd1‐611299 was most closely related to Beihai sobemo‐like virus 6 (YP_00933713), which was found in a mixed sample of superphylum Lophotrochozoa; the AAI of their RdRp sequences was 93.11% and their capsid proteins were also on the same branch (Figure 2B), but the AAI of the capsid protein sequences was slightly lower at 89.23%. Therefore, we think that these two viruses are different strains of the same virus. Tanwei sobemo‐like virus TWr1‐33874 clustered with Beihai sobemo‐like virus 7, which was found in phylum *Arthropoda*, but the AAI of the RdRp sequences was <30%. Like arthropods that feed on plants, bivalves such as oysters can also feed on aquatic plants or algae. Sobemoviruses were once considered to be plant‐specific viruses, but they have now been found in both arthropods and mollusks, providing a basis for the transformation of the virus in different trophic hosts [13].

Weiviruses are RNA viruses that were identified from invertebrates [9]. However, in the phylogenetic tree constructed with annotated ten capsid protein sequences and corresponding results of NCBI BLASTP, we found that Huangsha sobemo‐like virus HSd1‐611299, Beihai sobemo‐like virus 6, Beijing sobemo‐like virus 8, and Beihai sobemo‐like virus 10 clustered with the capsid proteins of Weiviruses (Figure 2B), whereas the phylogenetic tree constructed with the RdRp sequences did not contain any Weiviruses (Figure 2A). This finding implies that the capsid protein genes of sobemo‐like viruses and Weiviruses may have a common origin. The recombination between the capsid protein gene and RDRP provided clear evidence.

**Figure 3:**
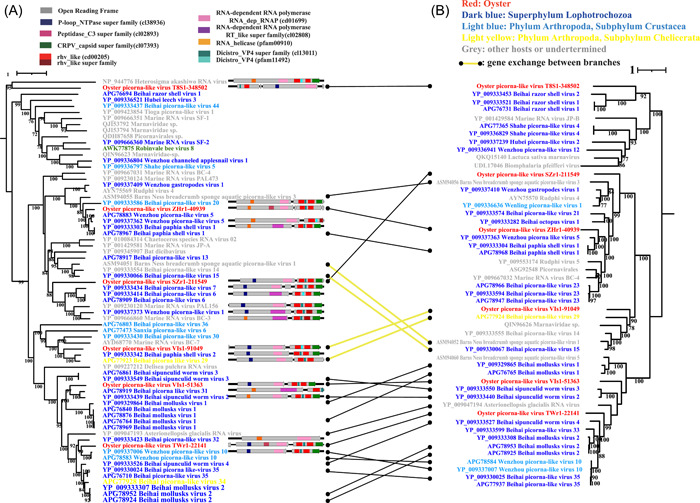
Comparison of the topological structure between the RdRp and capsid phylogenic trees of picornaviruses found in oysters. The trees were constructed using Iqtree (version 2.1.4) based on the RdRp (A) and capsid protein (B) sequences. ModelFinder was set to MFP and 1000 ultrafast bootstrap replicates were used. Bootstrap values >70 are shown.

*Picornavirales* were found to be the most abundant RNA viruses in coastal water [16, 17]. We also found six oyster‐associated *Picornavirales* viruses in this study (Figure 3). Oyster picorna‐like virus T8S1‐348502 was closely related to RNA virus (NP_944776) from *Heterosigma akashiwo* (*Rhaphidophyceae*), and oyster picorna‐like virus ZHr1‐40939 and oyster picorna‐like virus Vis1‐51363 were closely related to Wenzhou picorna‐like virus 5 (YP_009337362) and Beihai picorna‐like virus 31 (APG78919), respectively, which were found in mixed samples of superphylum Lophotrochozoa. The genetic relationship between oyster picorna‐like virus SZr1‐211549 and known viruses was distant. Oyster picorna‐like virus Vis1‐91049 was most closely related to Beihai picorna‐like virus 29 (YP_009337362) from the chelate subphylum *Arthropoda*, and oyster picorna‐like virus TWr1‐22141 was most closely related to Wenzhou picorna‐like virus 10 (APG785830) from *Arthropoda* (subphylum *Crustacea*). However, the AAI of the RdRp sequences among the six *Picornavirales* viruses and the unclassified *Picornavirales* viruses was <90; therefore, we think they are all new viruses.

When comparing the topological structure between the RdRp and capsid phylogenic trees of picornaviruses (Figure 3), although there is no evidence of recombination among the major clades, we still observed the gene exchange among some small branches (Figure 3, yellow lines). It is different from *Picornavirales* that the recombination was not found even among small branches on the phylogenetic tree of *Leviviridae* (Supporting Information: Figure S1).

*Leviviridae* is a kind of single‐stranded RNA virus that can infect a variety of Gram‐negative bacteria. *Leviviridae* shares the same core genome, which spans 3.4–4.3 kb and encodes a subunit of RdRp, mature protein, and coat protein [18]. We found three strains of *Leviviridae* viruses in this study, and all of their genomes encoded these three proteins (Supporting Information: Figure S1A). Among them, Taishan Levi‐like virus T4S1‐79710 and Huangsha Levi‐like virus HSd1‐59787, which were found in Guangdong, were most closely related to Beihai Levi‐like virus 28 (APG7701), which was found in Beihai, Guangxi, and Hubei Levi‐like virus 4 (APG77248), which was found in Hubei, respectively. However, the AAIs of their RdRp sequences were only 61.49% and 44.07%, respectively. We believe that the two strains belong to the newly discovered *Leviviridae*. Taishan Levi‐like virus T4S1‐672536, which was found in *Crassostrea hongkongensis* in Taishan, Guangdong, was closely related to Beihai Levi‐like virus 17 (APG77031), which was found in the *crustacean* subphylum of Beihai in Guangxi; the AAI of their RdRp sequences was 96.04% (Supporting Information: Figure S1A) and the AAI of their capsid protein sequences was 97.61% (Supporting Information: Figure S1B). Because the AAIs of these proteins were >95%, we think that these two viruses are different strains of the same virus.

### Ubiquitous and abundant oyster‐associated picobirnaviruses

*Durnavirales* are double‐stranded RNA viruses that can infect both vertebrates and invertebrates. In this study, we found six oyster‐associated *Durnavirales* viruses that clustered in a branch with unclassified *Picobirnaviridae* viruses (Supporting Information: Figure S2). Their genomes all contained a conserved RT_like superfamily domain (cl02808), but the number of ORFs was different (1 ≤ ORFs ≥ 4) (Supporting Information: Table S1). The AAI of the RdRp sequences of oyster‐associated RNA virus ZHd1‐112402, oyster picobirna‐like virus SZr1‐72709, and oyster picobirna‐like virus Yjd1‐298692 with the closest viruses was <60%. For oyster picobirna‐like virus ZHr1‐41827, oyster picobirna‐like virus Yjr1‐11446, and oyster picobirna‐like virus Yjr1‐2332 no closely related sequences were found in the NCBI nr database, and the AAI of the RdRp sequences between oyster picobirna‐like virus Yjr1‐11446 and oyster picobirna‐like virus Yjr1‐2332 was <90% (Supporting Information: Figure S3). Therefore, we think that the six viruses of *picobirnaviridae* found in this study are all new.

We calculated the abundance of these viruses in 54 oyster virus libraries from a variety of sources (Supporting Information: Table S2). Among them, oyster‐associated RNA virus ZHd1‐112402 was found in 24 libraries. The highest FPKM values were 36276.74 in library ChQZ1511Rb and 11132.95 in library ChQZ1511Ra. Oyster picobirna‐like virus YJd1‐298692 was found in 13 libraries, and the highest FPKM value was 3124.75 in library ChTW1511Ra (Supporting Information: Table S2). These two viruses are the most widely distributed and abundant of the 18 newly discovered RNA viruses, showing that picobirnavirus is an important member of the oyster.

Yanviruses are positive‐stranded or double‐stranded RNA viruses [9]. In addition to the virus found above, the oyster yanvirus‐like virus SZr1‐117762 was also found in this study. Although it was closely related to Wenzhou yanvirus‐like virus 2 (Supporting Information: Figure S4A), the average amino acid identity (AAI) of their RdRp sequences was only 68.57%. Therefore, we infer that the oyster yanvirus‐like virus SZr1‐117762 is new. Wenzhou yanvirus‐like virus 2 was derived from mixed samples of superphylum Lophotrochozoa, including *Bivalvia, Gastropoda, Cephalopoda, Polychaeta, Oligochaeta, Hirudinea*, and *Sipuncula Phascolosoma esculenta*, and *Sipunculus nudus*, which was composed of seven groups, and which was similar to the oyster sample from oyster yanvirus‐like virus SZr1‐117762. For oyster yanvirus‐like virus SZr1‐117762, although the RdRp domain was not detected by CDD, the RdRp sequence alignment results showed high AAI in the conserved RdRp domain (Supporting Information: Figure S4B), indicating that the RdRp of this virus had an atypical RdRp domain.

## DISCUSSION

Viruses are the most abundant biomasses in oceans, and mollusks, which are types of shellfish, are the largest group of animals in oceans. However, the intersecting field of shellfish and viruses is poorly understood. Virome sequencing has been widely used to analyze many biological and environmental samples, highlighting the potential of high‐throughput sequencing technologies for detecting new viruses [19]. In this study, we used virome technology to identify new RNA viruses in *C. hongkongensis* and found 17 new RNA viruses that showed only 30%‐70% similarity to their closest viruses, highlighting the genetic diversity of marine RNA viruses (Supporting Information: Table S1). However, two key technical issues remain to be solved in the classification and identification of new virus genomes. On the one hand, because the identification of viruses depends mainly on similarity searches in public databases, the ability to find and identify different or unknown viruses is highly restricted. On the other hand, the classification of RNA viruses is usually based on the highly conserved RdRp protein sequences. However, we found an asynchronous pattern between RdRp genes and capsid protein genes (Figure 1), and that recombination between the capsid protein gene and the RdRp gene may occur in RNA viruses (Figures 2 and 3). Therefore, using a single gene, such as RdRp, to infer the history of RNA viruses has major limitations.

Viruses from the same family or host species can infect species of different phyla or even different kingdoms at the same time. Such events are called host sharing and host switching. Studying host sharing and host switching events can help in the discovery of potential zoonotic viruses and prevent the occurrence of new epidemic diseases; for example, ranaviruses (family *Iridoviridae*) [20] isolated from reptiles, amphibians, and fish, and the cross‐species transmission of the novel coronavirus (SARS‐CoV‐2) [21, 22]. Our phylogeny results indicate that some of the viruses identified in this study may have host‐sharing characteristics; for example, the sobemo‐like virus was found in arthropods and mollusks, as well as in plants, and oyster picorna‐like virus T8S1‐348502 was found in *Picornavirales* clustered with *Heterosigma akashiwo* RNA virus (NP_944776). This may be due to the host transformation of *Heterosigma akashiwo* by oyster filtering of microalgae in water as food [23].

Viruses from oyster samples have been identified previously. For example, 26 new RNA virus genomes were assembled from the public transcriptome data of *C. gigas* and *Crassostrea corteziensis*. They included mainly *Dicistroviridae, Picornavirales*, herpes‐like viral family viruses, and the algae‐infecting viruses *Heterosigma akashiwo* and *Chaetoceros socialis* f. radians RNA virus 1 [10, 24]. Four RNA virus genomic fragments from oyster (*C. gigas*) samples have also been reported [13], and 33 novel RNA viruses were identified from mixed bivalve samples, including two oyster species *C. hongkongensis* and *C. ariakensis* [9]. The 33 viruses were distributed in *Narnaviridae* (nara‐like), Yanvirus (yanvirus‐like), Weivirus (weivirus‐like), *Totiviridae* (toti‐like), *Tombusviridae* (tombus‐like), *Picornavirales* (picorna‐like), and *Nodaviridae* (noda‐like). In addition, *Birnaviridae* RNA viruses were found in shellfish. A virus from Japanese pearl oysters (Pinctada fucata) presenting mass mortality was isolated, named “Marine birnavirus” (MABV) [25]. And aquabirnaviruses were reported from Geoduck clams (*Panope abrupta*), and littleneck clams (*Protothaca staminea*） collected in Alaska [26]. However, only one of the RNA viruses identified in this study had RdRp and capsid protein sequences that shared high AAIs with the RdRp and capsid protein sequences of these viruses (AAI > 90%); the other 17 viruses are quite different. Furthermore, we found two virus types, *Sobelivirales* (sobemo‐like) and *Leviviridae* (levi‐like), that had not been identified previously in oysters. In our previous mining of DOV data, we found that there were a large number of unclassified circoviruses in oysters [14].

RNA viruses found in oysters also exist in seawater and other marine animals. For example, the Picorna‐like viruses were found to be the most abundant RNA viruses in coastal water [16, 17] and were also found in marine fish [27] and shrimp [28]. Zhang et al. found *Duranvirales* and Sobemo‐like viruses in gastropods and crustaceans, respectively [13]. The white spot syndrome virus, the viral nervous necrosis virus, the marine birnavirus, and the viral hemorrhagic septicemia virus can be detected in both shellfish (including oysters) and seawater by nested PCR [29]. Although many studies have shown that the microbiota in oysters is mainly disturbed and influenced by the external environment [30, 31], it is significantly different from the environment. It indicates that the internal environment of oysters has a selective effect on their inner microbial community [14, 32]. All these data indicate that oysters have rich, diverse, and unique viral groups that are very different from the viruses found in marine invertebrates so far. Oysters can be regarded as repositories and vectors of marine viruses because of their filter‐feeding methods, low levels of immune defense mechanisms, and high‐density sessile lifestyles. Further studies on the community structure and function of bivalve viruses will greatly help in understanding their role in coastal microflora regulation, disease transmission, and the protection and restoration of coastal ecosystems.

## CONCLUSION

The characteristics of 18 RNA virus genomes found in oysters are summarized in this study. Seventeen of them are new virus species, which effectively expands the diversity of the oyster RNA viruses described so far. The common host transformation or host sharing of viruses in invertebrates, and the discovery that the capsid protein genes of sobemo‐like viruses and Weiviruses may have undergone recombination and exchange or have a common origin, have added to the understanding of oyster‐associated viruses.

## METHODS

### Sequence assembling and virus discovery

We constructed 54 oyster virus libraries from a variety of sources, including nine‐time points, seven sites (Qinzhou, Guangxi, Yangjiang, Zhuhai, Tanwei areas of Huidong, Lianjiang, Shenzhen), and two tissue types [14]. By virome sequencing of oysters (*Crassostrea hongkongensis*) cultured in many coastal areas of South China, we obtained approximately 2.5 billion reads [33]. Fastp (version 0.20.0) [34] was used to remove low‐quality sequences and adapters for quality control, and the reads were assembled into contigs using MEGAHIT (version 1.2.9) [35, 36]. DIAMOND (version 0.9.24.125) [37] was used to align and annotate the contigs with the National Center for Biotechnology Information (NCBI) nonredundant protein (nr) database as the reference. We classified the annotated sequences using MEGAN6 [38]. Finally, 18 virus genome sequences were identified as suspected RNA viruses and were screened for deep analysis.

### Open reading frame (ORF) prediction and annotation

ORFs were predicted in the eighteen virus genomes using Cenote‐Taker2 [39]. NCBI BLASTP [40, 41] was used to align the ORF sequences to the nr database with *e*‐value cutoff set as 10^−5^. The protein sequences with the highest consistency were inversely aligned with the virus genome sequences using NCBI tBLASTN [40, 41] to verify the integrity of the ORF predictions. We also carried out domain‐based searching using the NCBI Conserved Domain Database (CDD) [42, 43] with an expected value threshold of 0.001. SnapGene (version 4.3.6) was used to visualize the structure of the genomes.

### Similarity clustering analysis

We took the top 10 RdRp sequences and top 10 capsid protein sequences from the BLASTP results based on their total scores and used DIAMOND [37] to align them. Then, we used Gephi (version 0.9.2) [44] to construct clustering networks based on the scores.

### Phylogenetic tree construction based on RdRp and capsid protein sequences

We used MAFFT [45] for multiple sequence alignment, TrimAL [46] to remove ambiguous areas, and IQtree (version 2.1.4) [47] to build maximum likelihood phylogenetic trees based on the RdRp and capsid protein sequences. ModelFinder [48] was set to MFP (for ModelFinder Plus) and 1000 ultrafast bootstrap replicates were used. iTOL (version 6.5.2) (https://itol.embl.de) [49] was used for visualization.

### Analysis of the abundance of viruses

To calculate the relative abundance of each virus, we combined the 18 virus genome sequences and used the Salmon (version 0.13.1) [50] index command to generate a reference genome data set. Then, we used the Salmon quant command to map the clean reads of all the oyster virome libraries (PRJCA007058) one by one to the reference genome. Finally, we counted the number of mapped reads for each library. The relative abundance of each virus was calculated according to the adjusted FPKM (Fragments Per Kilobase of exon model per Million mapped fragments) as follows:

$$\text{FPKM}=\frac{\text{Genome reads}}{\text{Total reads}\times \text{Genome length}},$$

where genome reads is the number of mapped reads; total reads is the total number of reads obtained by sequencing, in millions; and genome length is the length of the genome, in Kb. Total FPKM is the sum of the FPKM of each library.

## AUTHOR CONTRIBUTIONS

**Peng Zhu**: Validation, Formal analysis, Investigation, Resources, Data Curation Visualization, Writing—Original Draft, Writing—Review & Editing. **Guang‐Feng Liu and Chang Liu**: Conceptualization, Methodology, Data Curation. **Li‐Ling Yang, Min Liu, and Ke‐Ming Xie**: Formal analysis, Investigation, Visualization. **Shao‐Kun Shi**: Investigation, Resources. **Mang Shi**: Conceptualization, Methodology, Validation, Writing—Review & Editing, Funding acquisition. **Jing‐Zhe Jiang**: Conceptualization, Methodology, Visualization, Resources, Data Curation, Visualization, Writing—Original Draft, Writing—Review & Editing, Supervision, Project administration, Funding acquisition. All authors have read the final manuscript and approved it for publication.

## CONFLICT OF INTEREST

The authors declare no conflict of interest.

## Supporting information

Supporting Information.

Supporting information.

## Data Availability

The data set supporting the results of this article has been deposited in the Genome Sequence Archive (GSA) under BioProject accession code PRJCA007058 [https://ngdc.cncb.ac.cn/gsub/submit/bioproject/subPRO010366/overview] and all RNA virus genetic sequences have been deposited in Genome Warehouse in National Genomics Data Center (NGDC) (Members and Partners 2021) under accession GWHBJCN01000000, GWHBJCM01000000, GWHBJCK01000000, GWHBJCJ01000000, GWHBJCI01000000, GWHBJCH01000000, GWHBJCG01000000, GWHBJCF01000000, GWHBJCE01000000, GWHBJCD01000000, GWHBJCC01000000, GWHBJCB01000000, GWHBJCA01000000, GWHBJBZ01000000, GWHBJBX01000000, GWHBJBW01000000, GWHBJBV01000000, GWHBJBT01000000, that are publicly accessible at https://bigd.big.ac.cn/gwh.
