# Supplementary material for: Novel RNA viruses in oysters revealed by virome
Source: Imeta. 2022 Nov 29;1(4):e65. doi: 10.1002/imt2.65 (PMC10989897; doi:10.1002/imt2.65)
Supplement: Supplementary file 1 — Supporting Information. [file IMT2-1-e65-s001.docx]

**Figure S1 Comparison of the topological structure between the RdRp and capsid phylogenic trees of levi-like viruses found in oysters.** The trees were constructed using IQtree (version 2.1.4) based on the RdRp (A) and capsid protein (B) amino acid sequences. ModelFinder was set to MFP and 1000 ultrafast bootstrap replicates were used. Bootstrap values >70 are shown.

**Figure S2 Maximum likelihood phylogeny of the “Partiti-Picobirna” clade found in oysters.** The maximum likelihood phylogenetic tree was constructed using IQtree (version 2.1.4) based on the RdRp amino acid sequences of Picorna-like viruses. ModelFinder was set to MFP and 1000 ultrafast bootstrap replicates were used. Bootstrap values >70 are shown.

**Figure S3 Phylogenetic analysis of the picobirnaviruses found in oysters.** The trees were constructed using IQtree (version 2.1.4) based on the RdRp (A) and capsid protein (B) amino acid sequences. ModelFinder was set to MFP and 1000 ultrafast bootstrap replicates were used. Bootstrap values >70 are shown.

**Figure S4 Maximum likelihood phylogeny of the yanviruses found in oysters.** (A) Maximum likelihood phylogenetic tree and genome structure of Yanviruses. The maximum likelihood phylogenetic tree was constructed using IQtree (version 2.1.4) based on the RdRp amino acid sequences of Yanviruses. ModelFinder was set to MFP and 1000 ultrafast bootstraps were used. Bootstrap values >70 are shown. The domains in the genome structure were annotated using the NCBI Conserved Domain Database. (B) Aligned RdRp amino acid sequences of two viruses, oyster yanvirus-like virus SZr1-117762 (DOV) and the Wenzhou yanvirus-like virus 2 sequence YP_009342252 (nr) as a reference. The red box marks the RdRp domain of the reference sequence.
